# BCR::ABL1-positive chronic myeloid leukaemia in a scenario of a remote diagnosis of JAK2-V617F-mutated polycythemia vera: a single patient experience with imatinib and ruxolitinib combination therapy

**DOI:** 10.1007/s00277-025-06700-8

**Published:** 2025-10-25

**Authors:** Paride Bartolucci, Federica Gigli, Valentina Tabanelli, Arianna Valeriano, Giuliana Gregato, Claudia Poletti, Francesco Bertolini, Enrico Derenzini

**Affiliations:** 1 Division of Haemato-Oncology, IEO, European Institute of Oncology IRCCS, Milan, Italy; 2 Division of Haematopathology, IEO, European Institute of Oncology IRCCS, Milan, Italy; 3 Laboratory of Haemato-Oncology, IEO, European Institute of Oncology IRCCS, Milan, Italy

**Keywords:** Polycythemia vera, JAK2V617 mutation, Leukemia, Myelogenous, Chronic, BCR-ABL positive, Imatinib mesylate, Ruxolitinib, Drug therapy, Combination, Myeloproliferative disorders, Case reports, Coexistence of JAK2, BCR-ABL mutations

## Abstract

We report the case of a 51-year-old male patient initially diagnosed with JAK2-V617F-mutated polycythemia vera (PV), who developed chronic phase BCR::ABL1-positive chronic myeloid leukemia (CML) 11 years later. The patient was treated with hydroxyurea and later with ruxolitinib (RUX) for PV. Following CML diagnosis, treatment with imatinib combined with RUX was initiated. Imatinib and RUX combination therapy proved to be safe and well-tolerated without major adverse events. After over one year of treatment, the patient maintained a complete cytogenetic response and a MR^2^ molecular response. Imatinib was switched to dasatinib due to the unsatisfying molecular response achieved. This case highlights the uncommon coexistence of JAK2 mutation and BCR::ABL1 translocation and supports the feasibility and safety of combining JAK2 inhibitors and tyrosine kinase inhibitors in such scenarios.

## Introduction

Myeloproliferative neoplasms (MPNs) are clonal hematopoietic stem cell disorders characterized by the proliferation of cells of one or more of the myeloid lineages. Depending on the presence or absence of a BCR::ABL1 translocation, they can be divided into BCR::ABL1-positive CML and BCR::ABL1-negative MPNs, respectively [1]. Significant numbers of BCR::ABL1-negative MPNs harbor an activating mutation in the Janus Kinase 2 gene (JAK2-V617F mutation) [2], and the JAK2 mutation and the BCR::ABL1 translocation are considered to be mutually exclusive. PV belongs to the BCR::ABL1–negative MPNs and is characterized by activating mutations in JAK2 (97% exon 14; 3% exon 12) leading to the proliferation of malignant hematopoietic stem and progenitor cells [3, 4]. Patients with PV present with erythrocytosis, variable degrees of disease-related symptoms (e.g. pruritus, night sweats, and fatigue), and an increased risk of both thromboembolic events and progression to myelofibrosis and acute myeloid leukaemia. A limited number of cases of CML occurring after an established diagnosis of BCR::ABL1-negative MPN have been described [5–11] and management strategies in this population are not defined. We report the case of a patient with JAK2-V617-mutated PV that several years after developed chronic phase BCR::ABL1-positive CML. No other cases of this kind are known to our center, which treats around a hundred patients affected by MPNs. Data regarding treatment response assessment and drugs association (JAK2 inhibitor and tyrosine kinase inhibitor) safety and effectiveness collected during a more than 1-year-long follow-up are reported.

## Case presentation

In October 2012, after a cerebrovascular accident that required prompt hospitalization, a 51-year-old man was first diagnosed with MPN. Complete blood count showed: white blood cell 13.350/µL, hemoglobin 17.4 g/dL, hematocrit 48.7% and platelet 630.000/µL. JAK2-V617F mutation detection on peripheral blood by polymerase chain reaction (PCR) was positive, meanwhile BCR::ABL1 fusion transcript resulted undetectable. The bone marrow examination was diagnostic of MPN, seemingly with morphological features suggestive of essential thrombocythemia. The stroke left no sequelae, the patient was started on clopidogrel for antithrombotic prophylaxis and hydroxyurea (HU) since December 2012 and he didn’t undergo further check-ups in the following 4 years. His medical history was not significant for other diseases.

Thereafter, the patient visited our clinic for the first time in December 2018 and complete blood count showed: hemoglobin 15.7 g/dL, hematocrit 47%, platelet 617.000/µL, white blood cell 7,140/µL under treatment with HU. A new bone marrow evaluation was performed: the specimen was hypercellular (~ 65%), showing trilineage hematopoiesis with a predominance of the erythroid lineage. Megakaryocytes were increased in number, exhibiting variable sizes and a heterogeneous distribution, including scattered and loosely clustered forms. Reticulin staining revealed grade I fibrosis. JAK2-V617F mutation was detected on peripheral blood by real-time quantitative polymerase-chain-reaction (RQ-PCR) with a value of 32%. These findings, together with values of hemoglobin and hematocrit at the upper limit of normal during treatment with HU, were all consistent with a diagnosis of PV. Ultrasound of the abdomen was normal and didn’t demonstrate splenomegaly. The patient continued with both HU and clopidogrel as he already did before. After 6 years of treatment with HU the patient began to experience mild cutaneous and gastrointestinal intolerance, the drug was gradually tapered until discontinuation and RUX 10 mg twice daily was commenced in May 2019. Complete blood count tests were repeated each 2 weeks and RUX proved to be reasonably effective in ameliorating both hemoglobin and hematocrit. Complete blood count after 6 months of treatment with RUX showed: hemoglobin 14.8 g/dL, hematocrit 44%, platelet 728.000/µL and white blood cell 7.460/µL. Patient kept being asymptomatic, in good general conditions, and RUX was well tolerated. On the basis of the good clinical response obtained and taken into account the improvement achieved on hematologic tests, since June 2021 a complete blood test each month was considered adequate for the follow-up. In May 2023, 4 years after that RUX had been started, blood tests values were: hemoglobin 13.7 g/dL, hematocrit 42%, platelet 581.000/µL and white blood cell 8.900/µL.

In October 2023 blood tests highlighted a leukocytosis of new onset (white blood cell count of 36.170/µL) with normal hemoglobin and platelet count. Peripheral blood smear showed leukoerythroblastosis (63% neutrophiles, 5% lymphocytes, 9% monocytes, 1% eosinophils, 6% basophils, 16% immature forms, of whom 13% immature granulocytes). Physical examination was negative for spleen enlargement. Flow cytometric immunophenotyping and next-generation sequencing (NGS) assays on peripheral blood were done. The immunophenotyping demonstrated the presence of promyelocytes, myelocytes and metamyelocytes accounting for the 30% of the total leukocyte population; increased CD34 + cells, with aberrant phenotype, equal to the 0.28% of leukocytes. NGS, performed using the *Oncomine*™ panel, showed JAK2 gene mutation, c.1849G > T, p.(Val617Phe) with a variant allele frequency (VAF) of 45.45% and BCR::ABL1 (p210 b2a2/b3a2) fusion transcript documented in 27.849 gene copies out of 27.296 control gene copies (102%).

**Fig. 1:**
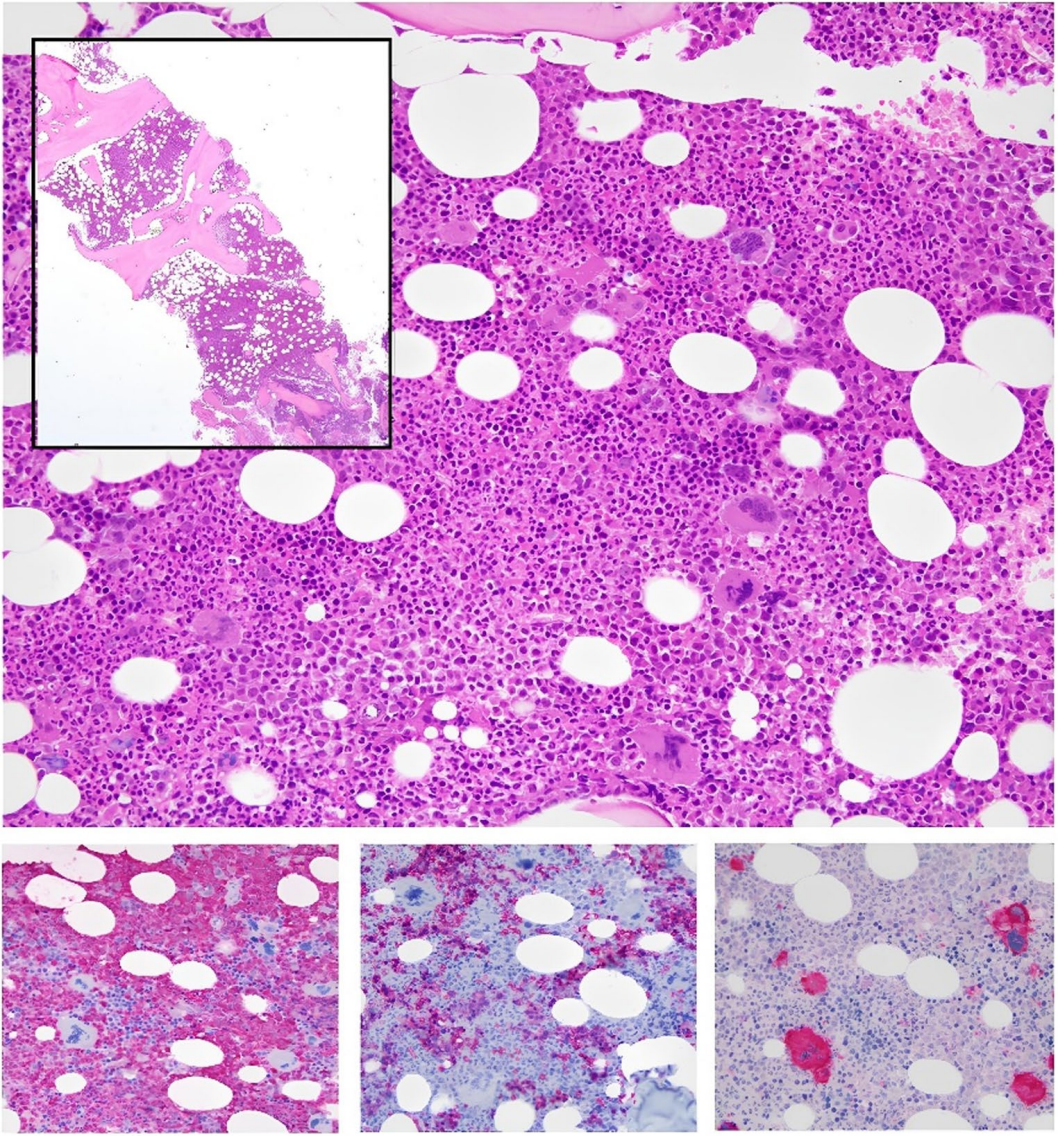
Bone marrow biopsy from 2023 demonstrating hypercellularity (inset) with granulocytic hyperplasia and full maturation (**A**, Hematoxylin & Eosin stain). Immunohistochemical analysis reveals an elevated myeloid-to-erythroid ratio (**B**, myeloperoxidase), and decreased erythroid precursors (**C**, Glycophorin C). Megakaryocytes were pleomorphic, ranging from small to enlarged forms (**D**, CD61 stain). Original magnification: 100×

These data suggested a diagnosis of CML of new-onset and the histology of bone marrow (Fig. 1) corroborated the suspect of Philadelphia-chromosome positive CML associated with Philadelphia-chromosome negative MPN (PV). Fluorescence in situ hybridization (FISH) on bone marrow confirmed the presence of t(9;22)(q34;q11.2) chromosomal translocation, involving the ABL1 gene locus on chromosome 9 long arm and BCR gene locus on chromosome 22 long arm, on the 71% of 200 interphasic nuclei analyzed. NGS on bone marrow blood showed the BCR::ABL1 fusion transcript in 23.842 gene copies out of 51.914 control gene copies (45.92%), and the JAK2 mutation c.1849G > T, p.(Val617Phe), with a VAF of 40%. These findings were all consistent with a diagnosis of chronic phase CML in a patient affected by PV. A well tolerated short course of cytoreduction with low dose HU was run and the tyrosine kinase inhibitor (TKI) imatinib was then safely started.

At the 3rd month of therapy with Imatinib the BCR::ABL1 fusion transcript was demonstrated on peripheral blood by RQ-PCR assay with an IS Ratio [(BCR::ABL1/ABL1) x 100 x conversion factor] of 4,42% (63.107 control gene copies number). Bone marrow blood NGS revealed the presence of BCR::ABL1 fusion transcript in 220 gene copies out of 7086 control gene copies (3.10%) and JAK2 mutation c.1849G > T, p.(Val617Phe) with a VAF of 31.83%; bone marrow blood cytogenetic assay highlighted normal male karyotype on 30 metaphases analyzed, corresponding to an obtained Complete Cytogenetic Response (defined as: no Philadelphia chromosome metaphases). The molecular response category at this 1 st time point (3rd month) for a BCR::ABL1 fusion transcript level ≤ 10% is defined *Optimal* [12]. In this case, the achievement of a Complete Hematologic Response (defined as: white blood count < 10 × 10^9^/L, platelet count < 450 × 10^9^/L, basophils < 5%, no myelocytes, promyelocytes, myeloblasts in the differential, spleen non palpable) has an inherent limitation due to the myeloproliferative nature of the other underlying disorder, namely PV on treatment with RUX presenting mostly with thrombocytosis, which acts as a confounding factor for what concerns the response assessment on blood counts.

A new disease response assessment was done at a 2nd time point at 6 months: the BCR::ABL1 fusion transcript RQ-PCR assay on peripheral blood corresponded to a molecular response^2^ (MR^2)^ (IS % Ratio 0.88, control gene copies number: 118.897). Cytogenetic assay on peripheral blood confirmed a Complete Cytogenetic Response. Bone marrow blood NGS demonstrated BCR::ABL1 fusion transcript on 26 gene copies out of 24.449 control gene copies (0.106%) and JAK2 mutation c.1849G > T, p.(Val617Phe) with a VAF of 33.94%.

At 9 months (3rd time point) the BCR::ABL1 fusion transcript level on peripheral blood, determined by RQ-PCR, matched to a MR^2^ (IS % Ratio 0.949, control gene copies number: 34.337). Cytogenetic on bone marrow blood was not evaluable. Bone marrow blood NGS results showed BCR::ABL1 fusion transcript on 33 gene copies out of 12.574 control gene copies (0.26%) and JAK2 mutation c.1849G > T, p.(Val617Phe) with a VAF of 35.30%. Bone marrow histology was repeated at this time point and it showed morphological abnormalities consistent with PV, meanwhile no evidence of CML was documented.

At 15 months, in February 2025, BCR::ABL1 measured on peripheral blood by RQ-PCR was 0.8% (IS % Ratio, 17.742 control gene copies number). In the light of the sub-optimal (MR^2^, *Warning* category) [12] response obtained, imatinib dose was increased to 600 mg.

1 month after imatinib dose increase, BCR::ABL1 on peripheral blood by RQ-PCR was 0.385% (IS % Ratio, 30.009 control gene copies number), MR^2^. The same response was obtained after 2 months of therapy: 0.82% (IS % Ratio, 42.367 control gene copies number), MR^2^. At this point imatinib was considered ineffective, in addition the patient started to complain episodes of dizziness after dose augmentation. Hence, the choice was to discontinue imatinib and dasatinib 100 mg was commenced. BCR::ABL1 tyrosine kinase domain mutations were investigated by NGS assay before treatment modification and resulted undetectable.

During this 1 year and 5 months of follow-up, blood tests showed stable values of both hemoglobin and hematocrit, with the first steadily being around 13–14 g/dL and the second around 38%. In contrast, platelets were constantly above the upper limit of normal, fluctuating between 600.000/µL and 800.000/µL No major thrombotic events occurred. Consistently, no significant increase on JAK2 allele burden has emerged.

## Discussion

CML can emerge in patients with Philadelphia-chromosome negative MPNs [5–11]. Herein, we describe the clinical picture of a patient harboring both a diagnosis of JAK2-V617F-mutated PV and a diagnosis of BCR::ABL1-positive CML, and one of the few available case reports of treating such a patient with a combination of imatinib and RUX.

RUX and TKIs (imatinib, dasatinib, nilotinib) given together have been used to treat patients with Philadelphia-negative MPNs and concomitant Philadelphia-positive CML [5, 10, 11]. No major adverse events are reported but in 2 cases RUX dose reduction or discontinuation of RUX and imatinib were necessary due to hematologic toxicity [11]. In our case drugs association seemed safe, well tolerated and devoid of major adverse events. After 17 months of treatment with imatinib and RUX the patient kept being asymptomatic, blood tests showed normal values of white blood cell count and both hemoglobin and hematocrit were steadily within limits but a thrombocytosis persisted. A complete cytogenetic response with low level BCR::ABL1 detectable by RQ-PCR was reached and maintained although matching to a sub-optimal molecular response (MR^2^, *Warning* category) [12]. In light of the limited available data of such an association [5, 10, 11], we gave priority to 1 st generation TKIs better safety profile [13, 14] and began with imatinib 100 mg without toxicity issues. RUX and the majority of TKIs, including imatinib, dasatinib, nilotinib and ponatinib [15], share a principal common metabolic hepatic pathway, mainly via CYP3A4 [16, 17], and imatinib is also described to be an in vitro CYP3A4 inhibitor [18] but no pharmacokinetic data of an interaction with RUX are reported. Hence, a try with imatinib 600 mg was made but, considered patient’s poor tolerance to dose increase and the unsatisfying response achieved, our choice was switching to dasatinib 100 mg. Dasatinib has higher activity compared to imatinib but is considered a less safe option due to a more common incidence of side effects [13, 14]. After one month of treatment with dasatinib no adverse events are reported, effectiveness is yet to be assessed. TKIs and ruxolitinib association has already been tested in patients affected by chronic phase of CML, in whom the addition of ruxolitinib to TKIs was associated with a significantly increased rate of patients reaching deep molecular response (MR^4.0^ and MR^4.5^) as compared to patients treated with TKIs alone [19]. The combination of the two classes of drugs could have a synergistic effect and contribute to achieving a deeper molecular response over time. In our case, compared to the results of the SWOG trial 1712 [19], the coexistence of the JAK2 mutation could impair this potential benefit. However, from this point of view, since a single-cell analysis couldn’t be performed, it’s not possible to know whether it’s a single neoplastic clone carrying both mutations or two distinct neoplastic clones, one harboring JAK2 mutation and one BCR::ABL1 translocation.

## Conclusion

This case highlights the uncommon coexistence of JAK2-V617F mutation and BCR::ABL1 rearrangement and supports imatinib and RUX combination therapy safety and feasibility in such scenarios. On the other hand, the optimal treatment regimen for patients with concomitant PV/CML is still unclear.

The authors did not receive support from any organization for the submitted work.

Written informed consent for publication of this case report and any accompanying images was obtained from the patient.

Ethics declaration: not applicable.

## Data Availability

No datasets were generated or analysed during the current study.
